# Work–Life Balance: Keep the Cycle Moving – Find a Purpose, Set Priorities, and Manage Time Well Then Reassess and Reset

**DOI:** 10.3389/fped.2015.00118

**Published:** 2016-01-05

**Authors:** Arun Saini

**Affiliations:** ^1^The University of Tennessee Health Science Center, Memphis, TN, USA

**Keywords:** work–life balance, work–life conflict, physician burnout, time management, mentorship

Life is like riding a bicycle. To keep your balance, you must keep moving. – Albert Einstein

We “the young physicians” are not immune to work and home stresses. On the contrary, we may be more prone to them (1). Dissatisfaction, depression, and burnout are common in physicians, especially in those with subspecialty training (2). Dyrbye et al. have found the lowest career satisfaction, greatest rates of work–life conflicts, and more significant depression in early-career physicians compared to middle- and late-career physicians (3). Despite the decrease in work hours, advances in the medical field, easier access to health information, and far superior connectivity to work and family members, young physicians continue to report high rates of dissatisfaction (4, 5). Physician dissatisfaction is an important issue and has significant consequences on various aspects of the modern health care system, including patient care, physicians’ well-being, and growth and viability of the medical field. Though there is growing recognition of physician dissatisfaction and its consequences, few interventional studies have been done so far to address this problem (6–8). To tackle this issue would require both individual and institutional efforts to seek strategies to help this vulnerable group.

As a pediatric intensivist, physician–scientist, spouse of a pediatric neurologist, and father of 4-year-old boy, I have experienced several conflicts with work–life balance up to this point in my life, and I am sure there will be plenty more in coming years. There were times when I questioned myself, “Is this worth doing”? I sat down to think and remembered these words:

Thou has knowledge declared to you; reflect on it fully and act as you like – Bhagavad Gita

In the hustle and bustle of busy work schedules and chores of daily life, young physicians often let themselves operate in autopilot, although we forget that to successfully run in autopilot requires some key elements, including a destination (purpose), a path to follow (priorities and time management), and ongoing monitoring of the current situation (reassess and reset) (Figure 1).

**Figure 1:**
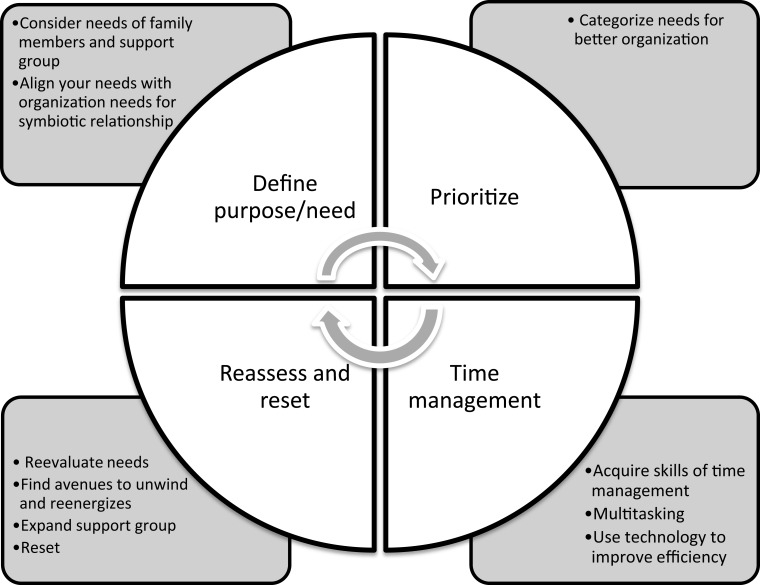
**Work–life balance: keep the cycle moving – find a purpose, set priorities, and manage time well then reassess and reset**.

The first step is to find the purpose in daily life to make it meaningful. Most young physicians feel dissatisfied and dispassionate in their work, because they do not see the need (purpose) behind it. Abraham Maslow proposed a “hierarchy of needs” in his milestone 1943 paper, “A theory of human motivation” (9). Maslow categorized needs as physiological, safety, love and belonging, self-esteem, and self-actualization. These needs are often depicted in the shape of a pyramid with the most fundamental needs at the bottom and the need for self-actualization at the top (9). These needs may not be universal and hierarchical as described by him and may vary from individual-to-individual. Marlow’s categorization helps prioritize needs in a more organized manner. We should also take into consideration the needs of our family members and support group.

On the larger front, it is important for both the young physicians and the health care institutes to make an effort to align their needs to create a more symbiotic relationship (10). Effective leadership and job satisfaction are two interrelated factors, which are fundamental to institutional success. With the changing economy there is a shift in the needs of health care institutes with focus on productivity, sustainability, and cost control. Many institutional leaders have adopted a transactional leadership style to accomplish institutional needs. This approach may have increased stress in young physicians, who may feel pushed to see more patients in less time, put in long work hours and to do extra documentation. Young physicians may perceive that their role is to realize institutional financial gain, which may contribute to job dissatisfaction. The health care institutes will be benefited from having leaders with transformational leadership style to lead and motivate their young physicians. The dimensions of transformational leadership include idealized influence, inspirational motivation, intellectual stimulation, and individualized consideration. This notion is not new to the health care system; in a systemic review of 53 studies, nursing staff was found to be more productive, encouraged, and felt satisfied with the nursing leaders who had transformational leadership style (11). Successful leaders are not only able to identify a larger purpose to help society but are also good in communicating their vision and goals to their subordinates. Institutional leaders should encourage innovative ideas, provide protected time, and seek resources to help young physicians. Also, they should attempt to integrate young physicians in each step of the planning of institutional goals and overall vision. Better understanding of institutional goals may help young physicians in aligning their needs to the institutional needs.

The second step is the time management, an essential skill for young physicians to acquire. I remember the days in medical school when I could afford to focus and spend days on an assignment. Now it is not possible. I struggle to multitasking research experiments, grant and manuscript writing, literature review, patient care, fellow education, my son’s school and social engagements – the list goes on. Unfortunately, young physicians receive very limited training in time management. Few medical publications are available to help improve time management skills for physicians (12–14). In an evidence-based review, Gordon and Borkan only found 15 publications describing time management techniques for physicians (14). The authors broadly classified effective time management strategies into four categories: (1) setting short- and long-term goals, (2) setting priorities among competing responsibilities, (3) planning and organizing activities, and (4) minimizing “time wasters” (14). I have acquired most of my time management skills by emulating my role models and mentors. We should also take advantage of advances in information and communication technology, such as email, virtual meeting, and online organizer to manage time well. My hope is that in the future, bodies such as the Accreditation Council for Graduate Medical Education (ACGME) will recommend more formal training in time management skills for physicians-in-training.

Young physicians who strive to excel often push themselves to both physical and mental limits. It is important to find avenues to unwind and reenergize to prevent chronic fatigue and depression from setting in. One way to accomplish this is by engaging in physical and recreational activities. Physicians in the US are found to be more physical activity in comparison to the general US population, but the degree of physically activity varies with career stage and geographical areas (15). By contrast, Iwuala et al. have found only 20.8% of health care providers in Nigeria had adequate physical activity, and 71.3% of them had body mass index above recommended values (16). Residents and fellows are found to engage in less physical activity than attending physicians and medical students, probably related to longer and less flexible work hours (17). The ACGME has implemented duty-hour regulations, which have decreased work hours for residents and fellows, but studies are needed to evaluate whether they have translated into less mental and physical fatigue in this group of physicians. We should take lessons from companies such as Google Inc. and Apple Inc., who have developed campuses with easy access to various recreational facilities for their employees. Health care institutes across the nation provide limited if any, access to recreational facilities to in-house physicians. Development of such facilities by health care institutes may provide an avenue to help improve both mental and physical health of physicians. For me, seeing my son growing from an infant to a toddler full of curiosity and enthusiasm has been a great escape from all the intensity at work. To keep up with my son’s energy, I started distance running and was able to run my first half marathon last year. The training has helped me improve my physical endurance and mental fitness.

Life is dynamic. We all go through major transitions and unexpected things happen. It is important to reflect to reassess one’s needs and reset priorities from time-to-time. In medical professional life, major transitions occur at the conclusions of medical school, internship, residency, and fellowship. Similarly, in family life, we go through major transitions such as relationships, marriage, childbirth, and deaths of family members. These transitions pose different challenges, and it is our responsibility to plan ahead for foreseeable transitions. It is vital to identify and acquire essential skills to make each of these transitions smoother.

Lastly, there are many ways to success, but no one can do it alone. We have to build a strong network of support groups, both at work and home to fulfill our needs. Most successful young physicians have many mentors to guide them through their career and life. Young physicians would immensely benefit from strong mentor–mentee relationships by learning from mentors’ valuable life experiences to prepare for major work and home transitions (18). However, successful mentoring requires both the mentor and mentee are committed and have interpersonal skills as well as support of the institution (19). I am fortunate to have many mentors so far to guide me in my so-far-nascent career and personal life.

In the week when I was writing this article, I also spent hours at the bedside while trying to manage a critically ill bone marrow transplant recipient patient with acute respiratory distress syndrome on extracorporeal membrane oxygenation (ECMO). The patient’s mother asked me many challenging questions, “Is she going to survive?” “How long should we continue the ECMO support?” or “Have you seen anyone survive something like this?” The patient’s mother was trying to take cues from my facial expressions and hoping for me to say something encouraging. My heart was full of emotion and mind was contemplating the words I should use. I wanted to be say something realistic yet hopeful. Somehow, I came up with, “I don’t know the future and I know odds are against us, but we would not know for sure if we don’t try.” Next few days were challenging, but the patient made a remarkable recovery and weaned off ECMO. The patient’s mother was beyond grateful to the entire team. It is moments like these that blur the distinction between “work” and “life.” Work seems purposeful and life seems balanced. We “the young physicians” need to remind ourselves that our true purpose is to take care of people when they are most vulnerable.

To conclude, for me, it is not about the work–life balance. It is about finding your purpose in life both at work and at home – and striving to fulfill it. The balance is in the motion, so keep the cycle moving.

## Conflict of Interest Statement

The author declares that the research was conducted in the absence of any commercial or financial relationships that could be construed as a potential conflict of interest.
